# How does depreciations management affect subsequent fiscal performance? The case of the Swiss cantons

**DOI:** 10.1186/s41937-017-0017-4

**Published:** 2018-02-06

**Authors:** Maxime Clémenceau, Nils Soguel

**Affiliations:** 1 Economic Advisory, EY, Al Saqr Business Tower, Sheikh Zayed Road, P.O. Box 9267, Dubai, United Arab Emirates; 2 Institut de hautes études en administration publique, University of Lausanne, IDHEAP, CH-1015 Lausanne, Switzerland

**Keywords:** Creative accounting, Depreciations management, Government deficits, Swiss cantons, Simultaneous equation model, C23, D73, H62, H72, H83

## Abstract

Creative accounting allows governments and, more particularly, finance ministers to somehow manage financial reports to achieve specific and possibly self-interested goals. It is usually used to hide deficits. It sometimes also helps to present financial performance as being more worrisome than it actually is. In that case, ministers press more than needed for lower expenses and a higher tax burden. This pressure is expected to tame deficits or increase surpluses over time. Using panel data relative to the 26 Swiss cantons over the period 1980–2013, we estimate econometrically how a political finessing technique like ‘depreciations management’ affects subsequent government expenses and revenues and thus subsequent financial performance.

## Background

What is the objective of accounting if not to provide various stakeholders with the information needed to take appropriate decisions? This is at least the theory supporting the international public sector accounting standards when they press governments to report their financial performance and position in a true and fair view, i.e. in a way that reflects the economic substance of transactions rather than just their legal form.^1^

A true and fair presentation of the situation is the standard against which all accounting practices have to be assessed. Any practice that would depart from this standard belongs in the category of creative accounting. Creative accounting is ‘the transformation of financial accounting figures from what they actually are to what preparers desire by taking advantage of the existing rules and/or ignoring some or all of them’ (Naser 1993, p. 2). As emphasised by Jones (2011), creative accounting is not necessarily a synonym of fraudulent action. Indeed, ‘managers will be happy to use creative accounting as they will be keeping to the letter, if not the spirit, of the law’ (p. 38). Actually, there exists a grey area between accounts that offer a fair presentation and those that are fraudulent. Within this grey area, accounts are managed in compliance with the rule of law. However, they do no longer offer a fair presentation of the situation. When accounts are managed, the apparent performance is modified (Stolowy and Breton 2004, p. 7–11). Subsequently, it becomes more difficult for stakeholders, and especially the legislature, to understand the government’s real fiscal performance or position and to take efficient decisions.

Creative accounting is mostly associated with damaging consequences. The recent crisis experienced by member countries of the European Monetary Union gives us evidence about the danger of such obfuscation. When reporting a performance that is artificially improved compared to the reality, cosmetic accounting reduces the need and the urgency of a structural fiscal adjustment. It leads to a level of indebtedness that is truly higher than what it would be should the performance have been reported in a true and fair way.

On the contrary, forms of creative accounting that underplay surpluses or even inflate deficits should have beneficial effects on the governments’ subsequent financial performance and indebtedness. For a government, reporting a surplus is politically risky. It would draw attention to the fact that taxpayers paid excessive taxes compared to the public services they received, or, to put it another way, that they do not receive an equivalent amount of public services with respect to the tax they pay. Lobbies or political parties may then claim for a tax reduction or increases in expenses.^2^ Such claims may in turn undermine the long-term fiscal balance, especially when the surplus was triggered by a favourable business cycle.

The political economy of deficits has investigated various influencing factors on a government’s financial performance. However, the specific impact of creative accounting has not been paid much attention so far. This is indeed the aim of this article: estimating the likely impact of previous creative accounting operations on the governments’ subsequent financial performance, paying particular attention to surplus-managing political finessing technique. The aim is also to identify through which channels previous creative accounting possibly impacts subsequent financial performance. Is it through a change in subsequent expenses or through a change in subsequent revenues? To the best of our knowledge, it has never been attempted before.

A large body of evidence supports the idea that European Union (EU) countries resorted to accounting manipulations, notably during the run-up to European Monetary Union (EMU). (Milesi-Ferretti and Moriyama 2006; Balassone et al. 2007). This literature also indicates that governments were encouraged by fiscal rules to manage their fiscal balances (Milesi-Ferretti 2004). Although Switzerland is no member of the EMU, Luechinger and Schaltegger (2013) did not rule out that deficits, at the cantonal level, were reduced through political finessing when they demonstrated that fiscal rules significantly reduced the frequency of deficits.

This paper contributes to this strand of literature, however from a different perspective. It expands Anthony’s argument (1985) that even if deficits are badly perceived, surpluses are not considered much better by politicians and citizens since a surplus highlights the notion that citizens paid too much in taxes or obtained too few public services. Therefore, a possible result of this situation is that politicians and citizens claim tax cuts or government expenses increases. The author suggests that states, local governments, or municipalities may be tempted to use conservative accounting practices to disguise surpluses or to report only small surpluses. For instance, Pilcher (2011) provides evidence that this kind of objective may be achieved using discretionary accruals. The author shows that local governments of the state of New South Wales in Australia rely on depreciation charges to do so. Stalebrink (2007) also demonstrates that Swedish municipalities use capital depreciations to manage reported financial performance. In his view, Swedish municipalities would increase capital depreciation during good economic periods and would do the opposite to dampen deficits. Although these studies found that creative accounting is also used to manage surpluses, they do not measure the quantitative impact of such practices on government financial performance. Precisely, this paper aims at measuring this very impact and broadens the spectrum of determinants that are dealt with by the political economy of fiscal deficits.

Many contributions focus on political determinants and notably on the political leaning of the government and parliament as recently done by Tellier (2006). Moreover, the political fragmentation of the government, measured as the number of political parties represented in a cabinet (Roubini and Sachs 1989) as well as the size of a cabinet, namely the number of spending ministers in a cabinet (Elgie and McMenamin 2008), has been investigated. At the same time, some studies analyse the impact of the solidarity between the legislative and the executive powers (Roubini and Sachs 1989). Furthermore, several studies identify a political budget cycle (e.g. Shi and Svensson 2006; Veiga and Veiga 2007). More recently, a new strand of literature, inspired by behavioural economics, is specifically interested in the role of the finance ministers in public sector financial management (Jochimsen and Thomasius 2014; Chatagny 2015). Apart from the political determinants, some studies investigate the impact of institutional factors on government deficits and indebtedness. In the Swiss context, they include the impact of budget rules or fiscal constraints (Feld and Kirchgässner 2008; Luechinger and Schaltegger 2013) as well as the effect of financial referendums and popular initiatives (Feld and Matsusaka 2003).

This paper investigates, among the institutional factors, how the budgeting and accounting technicalities can be used, especially by finance ministers, to secure the government’s financial performance. In this context, Chatagny and Soguel (2012) show that tax revenue misestimation in the beginning-of-the-year budget, due either to erroneous projections as a result of incomplete information or to strategic motives, significantly lowers the end-of-the-year deficits of the Swiss cantons. However, no paper has so far assessed how political finessing, and notably book entries that manipulate the amount of depreciation charges, affects future deficits (or surpluses).

To tackle this objective, this paper recourses to the methodology previously adopted by Krishnakumar et al. (2010)). In particular, it models simultaneously revenue and expenditure to take into consideration the structural restrictions imposed by the interdependence between revenue and expenses, since the fundamental principle of fiscal balance is rooted in the Constitution or the FMA of all Swiss cantons.^7^ Furthermore, it extends the time-span by a third compared to the previous empirical works dedicated to the Swiss cantons.

The 26 Swiss cantons provide a valuable setting for this empirical research. In Swiss cantons, several referenda have demonstrated the median voter’s preferences for balanced budgets and her aversion to deficits.^3^ The individual cantonal legislation, and in particular each cantonal Finance Management Act of Parliament (FMA^4^), reflects these preferences. Of course, the FMA differs from canton to canton as Swiss cantons maintain a high degree of autonomy in this area. However, almost all 26 FMA share a common conservative fiscal stance. This attitude finds concrete expression in the way expenses and revenues are reported. Most FMAs set out or tolerate certain political finessing. They especially allow the preparers to manage depreciation charges to inflate expenses and thus underplay surpluses where necessary or desirable; even though in doing so, the reported performance no longer reflects the canton’s exact balance, i.e. the balance that would reflect the sole economic substance of transactions. Although perfectly lawful, these kinds of entries clearly fall into the grey area mentioned by Stolowy and Breton (2004). The intercantonal conference of cantonal finance ministers explicitly admits that depreciations management is a tool for fiscal politics and involves the downside of creating hidden reserves in a way that lacks transparency (CFM 2008). According to the abovementioned and universally recognised definition given to creative accounting, depreciations management—just as earnings management—must be considered a form of a creative accounting since the account no longer offers a fair presentation of the situation. When earnings management manipulates the profit or the deficit of an organisation with book entries related to revenues (Healy and Wahlen 1999, p. 368), depreciations management uses book entries related to expenses.

The cantonal setting is also valuable since a common and detailed chart of account allows those with the necessary accounting skills and understanding to detect the book entries related to depreciations management. The main book entry that allows finance ministers to inflate expenses is the so-called additional depreciation charges (AD). With some insight and expert knowledge, the amount of AD that is charged to an accounting period can be spotted in the detailed annual general purpose financial report^5^ of the period, since a specific budget heading in the chart of accounts is used for AD.

Obviously, State auditors—just to mention them—are fully aware of the fact that these entries undermine the true and fair presentation of performance. However, the FMA passed by the Parliament sets the terms of reference for the auditor.^6^ It explicitly requires this latter to certify whether reports are law-abiding. Most noticeably, it does not require her to certify whether reports offer a true presentation. That paradox—knowing that the reported results might not present the truth but are nevertheless law-abiding—is an exclusivity that is worth exploiting and is exploited in this article.

Using panel data including information related to the 26 Swiss cantons for the period 1980–2013, we estimate two different models in order to measure the likely impact of depreciations management on the government’s financial performance. AD is our main explanatory variable, and it is constructed on the basis of the amount of ‘additional depreciation charges’ that were recorded at the end of each of these years and that we identified in each canton’s financial reports. The first model is a single-equation model where the financial performance (deficit or surplus) is the dependent variable. Then, we perform a simultaneous-equation model where the level of revenues and the level of expenses are simultaneously estimated. The advantage of the second approach is that it disentangles the respective possible effect of depreciations management on revenues and expenses. The results show that a positive and significant correlation exists between previous additional ‘depreciation’ charges and the subsequent level of revenues, whereas a negative correlation exists with the level of subsequent expenses, with a similar order of magnitude. Consequently, depreciations management seems to produce a significant positive impact on the future balance.

The remainder of the paper is organised as follows. In the 'Methods' section, we present the framework of our analysis from an accounting perspective, the quantitative importance of depreciations management in the Swiss cantons and both models to be econometrically tested. Results are reported and commented on in ‘Results and discussion’ section, followed by our final conclusion.

## Methods

### Statement of financial performance: key component of a government’s financial statements

When a government implements accrual accounting, instead of cash-based accounting, the statement of financial performance is the most scrutinised component of the financial statements.^8^ By comparing the operating revenues (R) with the operating expenses (E), its balance (*B* = *R* − *E*) shows whether citizens pay taxes that are equal to the public services they receive. The surplus (*B* > 0) or the deficit (*B* < 0) sparks the political discussion and captures both media and public attention. This is especially the case where a legal rule caps the deficit or even bans that possibility. In most Swiss cantons, the legislation governing public finance contains such a provision although at varying extents and magnitudes.

A government’s financial statements also include a statement of financial position (i.e. a balance sheet). This component attracts considerably less interest compared to what prevails in the private sector. However, among the assets and liabilities it presents, the stock of a government’s debt has lately become the noticeable exception. Eventually, the cash flow statement links both components by considering also the flow of cash from investing activities.

### Cash flow from operating activities and the change in debt level

Operating revenues (*R*) correspond by and large to inflows of cash resulting from operating activities. In terms of operating expenses (*E*), the correspondence with outflows of cash is weaker since not all expenses are disbursements. Typically, only part of the operating expenses (E*)—like wages, purchase of goods and services, and interest payments—drains cash. Other operating expenses such as depreciation charges (*D*) trigger no payment, meaning that they have no impact on cash.^9^ These book entries are purely accounting charges. In a true and fair view, the amount of the depreciation charge must accurately match the wear and tear and obsolescence of assets.

Therefore, *B* = *R* − (E* + *D*), since *E* = E* + *D*.

The cash flow from operating activities is equivalent to *R* − E* or *B* + *D*. It indicates how much cash remains after operating revenues are used to pay for cash-draining operating expenses. It also indicates how much cash is available to pay for capital expenditures and to pay down the government’s debt. For any given level of capital expenditure and depreciation, a reduction of *B* limits the amount of debt the government can pay down.

### Depreciations management to disguise performance and to maximise cash flow from operating activities

Accrual accounting increases the degree of technicality compared to cash-based accounting. As noted by Zimmerman (1977) or Giroux (1989), those who do not have expertise of accounting face many difficulties in dissecting the information provided by financial reports. The finance minister may rely on this asymmetry of expertise between himself on one side, the other members of the government (i.e. the spending ministers), and the members of parliament on the other side. The finance minister is all the more likely to do so when he has a legal obligation to balance the statement of financial performance or if he politically commits to lowering the debt.

A finance minister who would report a surplus may face requests for either enhanced services (i.e. an increase of E) or tax cuts in the near future (i.e. a reduction of R), or both. If those claims were effectively implemented, it would lower the balance over time and possibly lead to a deficit. In turn, this would lower subsequent cash flows from operating activities and the possibility of paying down debt.

In most cantons, the Financial Management Act of Parliament (FMA) offers the finance minister a strong conservative bias. The most common solution is the possibility—but not the obligation—to discretionarily inflate operating expenses by managing depreciations with ‘depreciation’ charges (AD).^10^ AD must not be confused with the ‘true’ depreciation charges (*D*) representing the very actual wear and tear and obsolescence of assets. The true depreciation charges are calculated using either the declining-balance method (constant depreciation rate applied on the residual book value) or the straight-line (constant rate applied to the acquisition cost of the asset). The rate is determined according to the useful working life of the considered asset. For each capital expenditure, the Parliament decides over the amount of the expenditure it authorises, the duration of the working life of the assets, and the depreciation charges that follows. Thus, there is no possibility for the executive branch of the government or for the finance minister to compromise on the amount of *D* that has to be recognised every year.^11^

By contrast, AD are depreciations just in name; they do not reflect any economic reality, any wear, tear or obsolescence of any asset. They are a pure accounting entry. They are named as such in the FMA, but they could also have been named ‘inflating expenses’ because it is their role: AD just inflate expenses without any corresponding outflow of cash (i.e. without any increase in E*). Following Stolowy and Breton (2004), they must be considered a form of creative accounting in spite of their legality, since the reported balance no longer truly presents the reality. For any given level of operating revenues, cash-draining operating expenses and true depreciation, AD deteriorate the reported balance of the statement of financial performance: *B*^R^ = *R* − (E* + *D* + AD) where *B*^R^ refers to the reported balance of the statement of financial performance, i.e. the one published in accounts and marred by AD. When AD > 0, *B*^R^ < *B*, *B* being the true balance.

The level of the cash flow from operating activities corresponds now to *B*^R^ + AD + *D*. However, its amount remains unchanged since *B*^R^ + AD = *B*. In this way, AD allows the finance minister to conceal, in whole or in part, a potential surplus without any change in the capacity to pay down debt or to invest.

### Quantitative importance of depreciations management in the Swiss cantons

Table 1 presents summary statistics pertaining to the use of additional depreciation charges in the Swiss cantons over the period of 1980–2012. It also presents the reported balance (*B*^R^) and the true balance (*B*), i.e. the reported balance corrected from the AD (*B* = *B*^R^ + AD). Data on additional depreciation charges (AD) and the reported balance (*B*^R^) were compiled directly by the authors from each of the annual financial reports of each of the 26 cantons. AD were identified using the specific budget heading under which they are classified according to the chart of accounts used by the Swiss cantons (heading 332 until year 2008 and 383 since then).^12^ Figures are expressed in real terms per capita.^13^ Given that Swiss cantons vary considerably in population and financial sizes, working with per capita figures allows us to control for this heterogeneity.

**Table 1:** Additional 'depreciation' charges, reported and true balance in real Swiss francs per capita—summary statistics per canton (1980–2012)

Canton	*N*	Additional 'depreciation' charges (AD)					Reported balance (*B*^R^)				True balance (*B* = *B*^R^ + AD)			
		*F*	Mean	Std. dev.	Min	Max	Mean	Std. dev.	Min	Max	Mean	Std. dev.	Min	Max
AG	19	9	4	9	0	27	83	178	− 147	439	81	805	− 3035	902
AI	34	28	168	159	0	780	50	60	− 37	192	239	194	− 79	800
AR	34	11	43	201	0	1173	1	526	− 1180	2546	51	233	− 458	827
BE	27	22	129	187	0	550	− 154	415	− 1054	300	− 117	499	− 2196	324
BL	33	27	95	198	0	941	60	231	− 263	693	191	302	− 257	822
BS	19	0	0	0	0	0	683	665	− 494	2083	384	1150	− 3151	1788
FR	18	1	14	59	0	250	251	813	− 131	3469	170	243	− 215	634
GE	28	1	63	333	0	1764	− 309	955	− 1726	1829	− 262	948	− 1719	1868
GL	34	12	74	151	0	648	41	372	− 551	1919	169	530	− 821	1912
GR	26	14	178	471	0	2438	196	559	− 794	1951	358	603	− 418	2201
JU	34	6	21	57	0	263	21	581	− 805	3144	− 147	273	− 899	246
LU	26	18	46	61	0	210	172	540	− 315	2603	126	269	− 378	686
NE	34	0	0	0	0	0	− 201	272	− 1342	90	42	241	− 655	416
NW	34	24	260	371	0	1559	49	136	− 231	510	256	355	− 239	1179
OW	28	17	154	197	0	717	161	713	− 224	3758	232	248	− 216	887
SG	17	0	0	0	0	0	127	207	− 273	659	44	300	− 504	636
SH	24	12	72	137	0	573	69	327	− 382	1343	132	270	− 411	767
SO	32	15	72	163	0	819	− 139	671	− 1910	2356	− 143	479	− 1019	793
SZ	27	27	143	102	17	373	123	460	− 929	1001	211	421	− 571	1105
TG	32	20	35	103	0	563	58	174	− 229	368	71	197	− 262	428
TI	34	5	64	207	0	901	11	442	− 965	914	20	478	− 988	1037
UR	30	18	552	635	0	2819	166	318	− 285	1131	627	631	− 304	1728
VD	34	12	42	97	0	472	− 127	399	− 796	535	− 60	458	− 871	705
VS	31	9	86	145	0	408	134	835	− 472	4504	167	356	− 416	842
ZG	34	31	286	193	0	766	463	471	− 174	1652	613	605	− 126	2370
ZH	32	5	2	6	0	16	12	390	− 1227	1079	− 10	369	− 1177	631
Overall	755	344	113	252	0	2819	61	520	− 1909	4504	129	514	− 3151	2370

Column *N* indicates the number of years during which each canton applied the common chart of accounts. Column *F* reports the absolute frequency of additional depreciation charges, i.e. the number of years at the end of which AD were recorded (thus *N* > *F*). The central panel shows the balance as it was reported by the cantons *B*^R^, i.e. the balance marred by additional depreciation charges. The right panel shows the corrected true balance *B*, i.e. the balance that would have been reported if AD had not been booked. Corrections were also made to suppress operations on cookie-jar reserves and exceptional operating revenues and operating expenses. Cantons are ranked according in alphabetical order

*Source*: Swiss cantons’ financial statements; compilation by the authors

Additionally, the column *N* indicates the number of years for which each canton applied the common chart of account and the column *F* reports the absolute frequency of additional depreciation charges (*N* ≥ *F*). For instance, the canton of Appenzell Innerrhoden (AI) was among the first to implement the common chart of account and has been using it for 34 years. Over that period, it has recorded additional depreciation charges in 28 of its 34 annual financial reports. The canton of St. Gallen (SG) was a late-adopter. However, it never recorded any AD in the 17 annual reports it has presented under the common chart of account. The cantons are ranked in descending order based on the average true balance over the considered period.

Twenty-three cantons out of 26 record AD at some point over the period. However, a strong heterogeneity exists among these cantons. The canton that registered the highest amount on average is the canton of Uri (UR) since it entered 552 Swiss francs (CHF) per capita on average compared to the canton of Zurich (ZH) which entered an average 2 CHF per capita. The canton of Uri is quite an extreme case since the second canton—the canton of Zug (ZG)—only entered 286 CHF per capita on average. Standard deviations together with minima and maxima and the absolute frequency also indicate a strong intertemporal variability.

The heterogeneity is also strong in terms of reported and true balances of the statement of financial performance. Nonetheless, the standard deviation appears to be smaller for reported balances in many cantons, suggesting that AD is effectively used to smooth reported balances. On average additional depreciation charges lower the surplus from + 129 CHF per capita down to a reported amount of + 61 CHF per capita. This points out the fact that Swiss cantons generally experienced a structural surplus which was partially managed thanks to the use of AD. This is at least true for cantons presenting a positive true balance (*B* > 0) on average between 1980 and 2013.

The picture is however quite different when paying attention to cantons presenting a negative corrected (or true) balance (*B* < 0) over the considered period. For those cantons (Bern-BE, Geneva-GE and Vaud-VD; with the exception of Solothurn-SO), inflating expenses with AD leads to an even larger deficit on average.

### Models and variables

In order to estimate the impact of previous creative accounting operations on the government’s financial performance, two approaches are used following Krishnakumar et al. (2010). A single-equation model directly estimates the impact of additional depreciations booked in the past on deficits and surpluses. Alternatively, a system of two equations, one for true revenues and one for true expenses, models the impact of previous creative accounting operations on the actual financial performance. Because of the conservative stance of the cantonal fiscal policy, models are estimated with regard to different possible outcomes. Model I considers all observation irrespective of the sign of the (subsequent) balance. Model II considers only the case where the (subsequent) true balance is positive (*surplus*). Model III is when the (subsequent) true balance is negative (*deficit*). Previous AD are especially expected to enhance (subsequent) surpluses.

### Single-equation model

The linear regression model explaining directly the impact of AD on the balance is expressed as follows:

$$ {B}_{i,t}=\alpha +\delta {\mathrm{AD}}_{i,t-1}+\kern0.5em \beta {X}_{i,t}+{\theta}_i+{\tau}_t+{\varepsilon}_{i,t} $$

where the dependent variable *B* is the cantonal corrected (true) balance as previously defined: *B* > 0 in a case of surpluses and *B* < 0 in a case of deficits. Our explanatory variable is AD(− 1), namely the first lag of the additional depreciation charges and *δ* is its associated coefficient. The first lag reflects the fact that (previous) creative accounting operations booked in the year *t* − 1 financial reports are expected to almost immediately influence the (subsequent) governments’ fiscal performance. Indeed, the government makes the year *t* − 1 fiscal result publicly available by the very beginning of year *t*. Therefore, AD(− 1) immediately limit administration units’ requests for additional budget appropriation even during fiscal year *t* and even though initial budget appropriations are insufficient. They also immediately exclude wishes for tax cuts starting in year *t* already.^14^

Then, *X* is the vector of control variables and β is the corresponding vector of coefficients. Furthermore, *θ* refers to cantonal fixed effects and *τ* to time fixed effects. The error term is represented by *ε*. Lastly, *i* and *t* denote the individual Swiss canton and year.

First, we control for the GDP growth rate: because of automatic stabilisers, it is expected to positively influence the revenues and negatively influence the expenses. Furthermore, we can reasonably expect that the unemployment rate (as a percentage of active population) deteriorates the governments’ financial performance by simultaneously increasing operating expenses and decreasing operating revenues. The composition of the population may also influence the governments’ financial performance. Notably, it is sometimes assumed (Feld and Matsusaka 2003) that a larger share of elderly in a canton’s population (older than 65 years) should generate higher healthcare and social expenses (elderly). But, on the other hand, this category of citizens may express a more conservative view on public finance. The governments’ political leaning also deserves a particular attention (leaning). Notably, right-wing governments have been shown to spend less than those that lean to the left (Tellier 2006). This variable reflects the average political leanings of cantonal governments.^15^ According to Roubini and Sachs (1989), fragmented governments are associated with higher public deficits. For that reason, the variable fragmentation captures the number of political parties represented in a government. The alignment between the executive and the legislative powers is also taken into account through the variable concordance, which is the share of seats in the parliament held by the parties represented in the government. According to Roubini and Sachs (1989), a misalignment between both powers should reduce the likelihood they reach agreements, which in turn is expected to result in excessive operating expenses. Furthermore, Valesco (2000) considers a government’s budget as a common good pulled by various interest groups. Thus, a large amount of spending departments (i.e. spending ministries, by contrast with the finance ministry) within a cantonal administration should lead to higher operating expenses. Departments should therefore be negatively associated with the level of public balances. Then, according to several authors among which Shi and Svensson (2006), the electoral cycle prompts an increase in government expenses and a reduction of revenues during election years in an attempt by incumbent politicians to raise their chances of being re-elected. Election is a dummy variable taking the value of 1 during election years and 0 otherwise. Governments also have to cope with institutional constraints. Among these constraints, popular initiatives and referendums, including a financial referendum, are typical institutions of Switzerland’s direct democratic system at the cantonal level. According to Feld and Kirchgässner 2000, financial referendums increase citizen scrutiny and prompt governments to keep expenses under the limit above which a project is sent to ballot box. The limit being more or less restrictive depending on the canton, the dampening effect varies. As for popular initiatives, they allow citizens to suggest changes in legislation. Suggestions may lead either to an increase or to a decrease of government expenses. The ease with which both tools can be used is measured with the index put forward by Stutzer and Frey (2000). Finally, previous works (Feld and Kirchgässner 2008; Luechinger and Schaltegger 2013) indicate that fiscal rules force governments to lower deficits and to show a positive structural balance over time. The variable reflecting the stringency of fiscal rules is the one proposed by Luechinger and Schaltegger (2013), where the value is 3 in case the rule of the canton is among the most stringent, 2 where the rule is fairly stringent, 1 for the least stringent, and 0 otherwise (no rule). We also control for the misestimation of tax revenue, which refers to the difference between the forecasted amount of tax revenue and the reported tax revenue. As shown by Chatagny and Soguel (2012), finance ministers in the Swiss cantons generally strategically underestimate tax revenue during the budgeting process, possibly to put public expenses under pressure. Underestimating tax revenue (misestimation < 0) should therefore decrease deficits (therefore β < 0). With balance (−1), we eventually control for the fact that *B* is not independent from its previous level. It may suffer from temporal inertia in particular because governments frequently resort to incremental budgeting.^16^

In order to ensure the significance and the consistency of our results, some elements must be considered. First, Swiss cantons are quite heterogeneous and this variability affects also the size of their various budgets. Expressing the variables per capita does not fully prevent the error terms to suffer from heteroskedasticity, as shown by the Breusch-Pagan/Cook-Weisberg test.^17^ Secondly, despite the inclusion of a lag on the dependent variable in the model, the Wooldridge test for autocorrelation reveals the presence of a first-order serial correlation. Nevertheless, the Arellano-Bond test does not confirm the presence of a second-order autocorrelation. Thirdly, as all cantons belong to the same country, error terms are possibly contemporaneously correlated. Fourthly, fixed effects allow the model to capture differences that can be hardly measured or observed. The Breusch and Pagan test as well as the Hausman test validate this strategy. Furthermore, the inclusion of cantonal fixed effects is more relevant than random effects when considering the whole population, i.e. the 26 Swiss cantons. However, the inclusion of cantonal fixed effects increases the risk of multicollinearity. Fifthly, our panel is unbalanced as not every Swiss canton introduced the recommended accounting model simultaneously. Nevertheless, it does not cause any econometric concern since the time of introduction varied randomly among them. Therefore, the reason why data are missing for some cantons is exogenous. Finally, the time series is longer than the cross section: *T* = 33 years (1980–2012) compared to *N* = 26 cantons.

We report here only the results obtained with the Panel Corrected Standard Error (PCSE) estimator. This estimator has the advantage of correcting heteroskedasticity and contemporaneous correlation. It also deals with first-order autocorrelation. Furthermore, the panel needs not to be balanced, and the estimator efficiently works when the cross section is smaller than the time series. Cantonal and time fixed effects are also included with the PCSE estimator in order to take into account regional and historical specificities not captured by control variables.^18^ Given the characteristics of the dataset, alternative estimators were utilised to make sure that the results are robust: an OLS estimator including cantonal fixed effects, where error terms are corrected with the White procedure; an estimator proposed by Baltagi and Wu (1999) which fits a cross-sectional time series regression model when the error terms are first order correlated (REGAR); a two-stage least square (2SLS) estimator where heteroskedasticity and serial correlation are taken into account by applying the White correction; and the system-GMM procedure which allows standard errors to be robust to heteroskedasticity and patterns of autocorrelation within individuals.

Some regressors may be potentially endogenous. Therefore, some valid instruments must be used to avoid biased and inconsistent estimated parameters.^19^ The tax revenue misestimation may be endogenous. Indeed, since the error is computed as the difference between forecasted and actual tax revenue, some simultaneity may appear between the variables’ balance and misestimation (Chatagny and Soguel 2012). The first difference of the tax revenue misestimation is used as an instrument. Also, the fiscal institutions themselves may also be endogenous since their stringency could be modified depending on the cantonal financial performance. Following previous works (Feld and Matsusaka 2003; Martin 2008), we consider the financial referendum, the popular initiatives, and the fiscal rules as potentially endogenous. Since our data do not exhibit second-order autocorrelation, we use the second lagged value of these variables as instruments.

### Simultaneous-equation model

Obviously, the true deficit or the surplus in the statement of financial performance is the difference between the true operating revenues *R* and the true operating expenses *E*. Therefore, using a simultaneous-equation model allows us to distinguish through which channel—revenues and/or expenses—additional depreciation charges eventually affect the (subsequent) financial performance. Following the seminal idea of Lowry and Alt (1994), Krishnakumar et al. (2010) presented a first attempt to simultaneously model revenue and expenditure. Chapman and Gorina (2012) elaborate the concept further. On the basis of the public choice literature and on a general model of government budget (which is not reproduced here), they state that ‘any estimation of expenditure and revenue equations must involve the use of the simultaneous equation framework’ (p. 23). Benito et al. (2013) also use the assumption of simultaneity. Essentially, while the level of operating revenues originating from various sources—including taxation and fiscal equalisation—will mostly determine the level of operating expenses, the latter should also influence the operating revenues to be collected by the government in order to achieve a particular level of public service provision. As a consequence, operating revenues and operating expenses are explanatory endogenous variables. Thus, the model comprising two equations can be written as follows:

$$ {R}_{i,t}={\alpha}^R+{\delta}^R{\mathrm{AD}}_{i,t-1}+{\gamma}^R{E}_{i,t}+{\beta}^R{W}_{i,t}+{\theta}_i^R+{\tau}_{\mathrm{t}}^R+{\varepsilon}_{i,t}^R $$

$$ {E}_{i,t}={\alpha}^E+{\delta}^E{\mathrm{AD}}_{i,t-1}+{\gamma}^E{R}_{i,t}+{\beta}^R{Z}_{i,t}+{\theta}_i^E+{\tau}_t^E+{\varepsilon}_{i,t}^E $$

where the *α*^*R*^ and *α*^*E*^ represent the intercepts, *γ*^*R*^ measures the marginal effect of expenses on revenues and *γ*^*E*^ the marginal effect of revenues on expenses.^20^ *W* and *Z* are the sets of control variables explaining operating revenues and operating expenses, and *β*^*R*^ and *β*^*E*^ are their associated coefficients. Although they include the same set of control variables, *W* and *Z* are still different as they each include the lagged of the dependent variable of their respective equations. To test whether *R* and *E* are Granger-caused, *W* also include the lagged value of *E* and *Z* also include the lagged value of *R*. Then, *θ* and *τ* are two-way fixed effects, and *ε*^*R*^ and *ε*^*E*^ are the error terms. *δ*^*R*^ and *δ*^*E*^ are the coefficients associated to our variable of interest, AD.

The different features of the model must be considered to produce consistent estimates. As for the single-equation framework, heteroscedasticity is treated using White correction for standard errors. What is more, some regressors in *W* and *Z* are correlated with *ε*, i.e. endogenous. Additionally, because *R* and *E* are simultaneously determined, they are endogenous too. Hence, such endogeneity requires the use of an estimator of the IV class. With the system of simultaneous equations, $E\left[{\varepsilon}_{i,t}^R{\varepsilon}_{i,t}^E\right]=0$ does not hold and allows the efficiency to be increased by using an estimator that also takes into account the correlation between error terms. As the set of instruments is the same across both equations, we use the three-stage least square (3SLS) estimator.

## Results and discussion

### Single-equation model

Table 2 reports results from the single-equation model explaining the financial performance of the overall sample (model I). It also reports results for both subsamples: when the balance turns out to be a surplus in the near future (model II) or a deficit (model III). AD(−1) shows the expected positive relationship: inflating charges with 1 additional CHF AD per capita in the past helps to improve the fiscal performance by 0.259 CHF (model I) and especially to generate and increase a surplus by 0.190 CHF (model II). As for reducing the near future deficit (model III), the coefficient, although positive, is not significant. More generally, according to the *R*-squared, the specification is less effective in explaining deficits than in explaining surpluses.

**Table 2:** Results for the single-equation model

	Overall sample	Subsamples	
	Model I	Model II	Model III
		Surpluses only (*B* > 0)	Deficits only (*B* < 0)
AD(− 1)	0.259***	0.190***	0.169
	(0.076)	(0.067)	(0.140)
Growth	12.835	4.075	11.615
	(8.715)	(6.890)	(12.364)
Unemployment	− 40.515	− 36.414	− 18.726
	(27.800)	(25.272)	(31.027)
Elderly	− 41.152*	− 43.221***	6.601
	(24.233)	(14.872)	(25.877)
Leaning	− 176.717***	− 77.532*	− 147.956**
	(56.748)	(40.538)	(63.851)
Fragmentation	− 15.799	− 29.366	17.861
	(42.744)	(29.361)	(47.090)
Concordance	− 62.071	− 144.951	211.956
	(173.191)	(140.321)	(258.737)
Departments	− 3.934	− 3.181	10.987
	(11.357)	(9.977)	(13.955)
Election	3.995	39.070	− 42.618
	(37.079)	(26.965)	(41.752)
Initiative	103.349	175.484***	− 25.178
	(84.209)	(61.002)	(118.961)
Referendum	− 42.176	− 56.754	68.208**
	(35.967)	(34.767)	(34.664)
Rules	− 5.540	− 19.214	− 24.714
	(47.459)	(25.928)	(58.857)
Misestimation	− 0.439***	− 0.426***	0.270
	(0.129)	(0.069)	(0.173)
Balance(− 1)	0.262***	0.220***	− 0.003
	(0.084)	(0.033)	(0.075)
Constant	1511.481***	999.567**	− 388.93
	(576.440)	(429.977)	(744.623)
Cantonal FE	YES	YES	YES
Time FE	YES	YES	YES
R-Squared	0.488	0.596	0.513
Joint, *p* value	0.000	0.000	0.000
N	729	475	254

(1) Dependent variable: cantonal corrected (‘true’) balance in real terms per capita at time *t*; (2) cantonal fixed and time fixed effects are considered; and (3) parameter values appear without brackets and the standard deviation within. Asterisks denote the level of significance of parameter values: *** indicating significance at 1% level, ** at 5% level and * at 10% level

A potential reverse causal effect of balance on AD might exist if we postulate that expectations about future budgets have an impact on AD. For example, the finance minister could use creative accounting more extensively if he expects larger deficits in the future. We cannot entirely rule out this risk, notably because it proves impossible to produce convincing instruments. Thus, causality cannot be indubitably claimed.

Regarding significant control variables, the models confirm some of the findings of the existing literature, while shedding additional light by segmenting into cases of surplus and deficits. Surplus tends to be lower when the share of elderly is larger in the population (model II). Balance tends to be improved and the deficit to be smaller when governments lean more to the right (leaning) (especially model III). Surplus tends to be larger when it is easier to use the right to petition (initiative, model II), whereas deficit tends to be inferior when it is easier to have recourse to the right of referendum (model III). The tax revenue misestimation also strongly increase surpluses (model II), typically in case of underestimation (misestimation < 0), without having a significant impact on deficits. The models also confirm that a path dependency exists, but that is especially the case for surpluses: the larger the previous surplus, balance (−1), the larger the (subsequent) surplus (model II). Noticeably, such path dependency does not emerge in case of previous deficits (model III). Although some of the control variables are individually not significant, joint tests of significance show that the coefficients are jointly significant.^21^

### Simultaneous-equation model

Table 3 below reports results from the simultaneous-equation model explaining the financial performance as the balance between revenues and expenses. Inflating previous expenses with AD significantly correlates both with (subsequent) operating revenues and with operating expenses. As expected (subsequent) revenues are positively correlated and expenses negatively. The correlations are mostly significant with the overall sample (model I) and in case of surplus (model II). However, the relationship appears less significant in the case of operating expenses when considering the subsample pooling the deficit situations only (model III).

**Table 3:** Results for the simultaneous-equations model

	Overall sample (I)		Surpluses only (II)		Deficits only (III)	
	Revenues	Expenses	Revenues	Expenses	Revenues	Expenses
AD(− 1)	0.207***	− 0.262***	0.221***	− 0.271***	0.268**	− 0.265*
	(0.056)	(0.058)	(0.051)	(0.045)	(0.107)	(0.153)
Growth	3.659	− 5.556	− 0.192	− 3.688	15.911*	− 14.975
	(6.436)	(6.841)	(6.41)	(5.979)	(9.156)	(13.194)
Unemployment	− 56.984***	22.964	− 35.629	23.669	21.437	− 31.884
	(20.237)	(22.095)	(25.671)	(23.867)	(28.695)	(41.494)
Elderly	− 22.386	31.019*	− 20.099	41.534***	− 26.254	− 10.134
	(16.387)	(17.109)	(16.591)	(14.863)	(29.267)	(44.115)
Leaning	− 148.549***	120.13***	− 68.121	91.283**	− 115.571**	71.400
	(36.736)	(40.465)	(44.633)	(41.462)	(48.061)	(73.771)
Fragmentation	− 46.035	38.552	− 59.238*	33.214	5.141	3.870
	(28.547)	(30.422)	(29.314)	(27.369)	(41.780)	(59.581)
Concordance	− 24.939	− 38.935	− 161.682	108.808	− 143.424	5.217
	(131.669)	(140.563)	(135.00)	(125.368)	(197.674)	(286.535)
Departments	12.598	14.331	9.366	6.341	12.147	− 3.524
	(10.512)	(11.133)	(12.031)	(11.235)	(11.761)	(17.287)
Election	7.102	− 2.610	32.450	− 30.577	− 40.910	27.665
	(26.226)	(28.006)	(27.535)	(25.594)	(31.615)	(45.538)
Initiative	110.642	− 101.956	209.760*	− 230.671**	− 514.333**	696.310**
	(111.601)	(120.518)	(105.122)	(98.630)	(201.913)	(281.781)
Referendum	24.247	79.232	− 23.316	94.424**	282.286***	− 270.813**
	(51.705)	(55.696)	(51.032)	(47.515)	(82.116)	(116.093)
Rules	− 32.723	23.074	− 38.696	45.861	50.581	− 158.574*
	(40.642)	(43.582)	(48.987)	(45.083)	(56.873)	(82.777)
Misestimation		− 0.491***		− 0.262***		− 0.164*
		(0.118)		(0.101)		(0.084)
Revenues		0.396***		0.681***		0.810***
		(0.064)		(0.054)		(0.103)
Expenses	0.584***		0.829***		0.878***	
	(0.049)		(0.040)		(0.036)	
Revenues(− 1)	0.315***		0.145***		0.063**	
	(0.032)		(0.027)		(0.030)	
Expenses (− 1)		0.396***		0.202***		0.156***
		(0.473)		(0.038)		(0.055)
Revenues (− 3)		0.045***		0.002		0.035
		(0.017)		(0.013)		(0.035)
Expenses(− 3)	0.074***		0.058***		0.054**	
	(0.016)		(0.014)		(0.021)	
Constant	691.67	− 71.398	− 39.678	− 153.464	1270.877	− 1381.556
	(561.543)	(594.001)	(572.274)	(527.073)	(904.745)	(1306.944)
Cantonal FE	YES	YES	YES	YES	YES	YES
Time FE	YES	YES	YES	YES	YES	YES
*R*-Squared	0.988	0.987	0.994	0.995	0.990	0.990
Joint *p* value	0.000	0.000	0.000	0.000	0.000	0.000
N	695	695	451	451	244	244

(1) Dependent variables: true operating revenues and true operating expenses in real terms per capita at time t; (2) cantonal fixed and time fixed effects are considered; (3) three-stage least square (3SLS) estimator; (4) Parameter values appear without brackets and the standard deviation within. Asterisks denote the level of significance of parameter values: *** indicating significance at 1% level, ** at 5% level and * at 10% level

In view of this, the improvement in subsequent financial performance stemming from (previous) additional depreciation charges seems to come through both channels: a support for (subsequent) tax revenue and a pressure on (subsequent) operating expenses. The effect is almost equal, with 1 additional CHF AD per capita corresponding with subsequent revenues to be 0.207 CHF higher and subsequent expenses to be 0.262 CHF lower (model I). Thus, the combined effect improves subsequent financial performance by about 0.469 CHF per capita. This impact is slightly larger than the one estimated from the single-equation model. However, this measure is more precise since the simultaneous-equation model partials out more precisely the effects of the control variables on expenses and revenues.

As for the control variables, the previous findings are broadly confirmed, especially with elderly, leaning, initiative, referendum and misestimation that correlate either with operating revenues or operating expenditure and therefore ultimately with the financial performance. With a level significance of 10%, rules correlates—as expected—negatively with operating expenses.^22^ The already mentioned path dependency appears present on both sides of the budget. Consistent with the existing literature on incremental budgeting, (subsequent) revenues and expenses depend partially—but not fully—from their (previous) level (− 1). Table 3 also shows that coefficients on expenses (− 3) are statistically significant in the revenues equations (in all three models) and that revenues (− 3) is statistically significant in the expense equation (at least in model I). Thus, it seems that revenues are Granger-caused by expenses and vice-versa.^23^

### Robustness checks

On top of estimating the single-equation model using alternative estimators, several robustness checks were performed to ensure the reliability in the results. A necessary precaution followed the splitting of the overall sample into two subsamples. The subsample pooling the surplus situations only is censored (*B* > 0) and so is the subsample pooling deficit situations only (*B* < 0). Such truncation at zero may lead to inconsistent linear regressions estimates. Thus, we estimated model II and model III using the Tobit estimator. We also tested the models excluding insignificant independent variables. Additionally, we excluded the canton of Uri (UR) from the data set since UR shows particularly high amounts of additional depreciation charges. Similarly, we excluded the cantons of Basel-Stadt (BS), Neuchâtel (NE) and St. Gallen (SG) since their government did not resorted to AD over the considered period. We re-estimated the various models without the concerned cantons. Whatever the robustness check, the sign of the coefficient and the significance of the explanatory variables and especially of our variable of interest (AD) remain stable.^24^

## Conclusions

This paper considers the extent to which creative accounting operations affect the fiscal performance. Governments and especially finance ministers are able to somehow manage reports. What comes immediately to mind in a general sense are the creative accounting operations used to hide deficits. This is certainly true in most instances. However, certain operations may also be used to disguise surpluses and to present a government’s financial performance as being more worrisome than it actually is. Compared to the spending minister or to members of parliament, the finance minister has a specific expertise in financial issues that makes it quite easy for him to resort to such practices. And as opposed to spending ministers, his personal agenda is to avoid (or lower) public deficits and indebtedness by putting operating expenses under pressure and by pressing for higher taxes than otherwise wanted.

We used panel data about the 26 Swiss cantons over the period 1980–2013. This setting is interesting since the individual cantonal legislation sets out or tolerates depreciations management operations especially designed to inflate expenses—with the so-called additional depreciation charges—and thus to worsen the financial performance in a way to specifically manage surpluses. Two different models of fiscal performance were estimated. The first model relies on a single equation with the balance (deficit or surplus) as the dependent variable. In the second model, the impact on the balance is indirectly considered since a set of two simultaneous equations is used: one with the operating revenues as the dependent variable and the other with operating expenses as the dependent variable. This simultaneous strategy allowed us to disentangle the respective effect of depreciations management on revenues and expenses and therefore provide more precise estimations compared to the single-equation model. The econometric results are based on several estimators and proved to be robust. They confirm that a positive and significant correlation exists between previous additional depreciation charges and the subsequent level of revenues, whereas a negative correlation exists with the level of subsequent expenses, with a similar order of magnitude. Consequently, depreciations management seems to produce a significant positive impact on the future balance.

From a policy viewpoint, these findings should be set in relation to the ongoing international tendency towards implementing more stringent accounting standards in order to foster governmental transparency. Obviously, governments must be prevented from cooking their books to disguise an unsustainable financial situation. However, in countries like Switzerland where budgetary policy follows a rather conservative stance, the possibility given to the government to use political finessing to somehow manage the fiscal performance allows it to sustain this stance and to fulfil the requirement of the fiscal rules when they exist. Depreciations management fosters a kind of elbow room to ensure structural surpluses or to prevent structural deficits. If accounting standards would rule it out, the structural surplus would be reported *en pleine lumière,* generating claims for tax cuts or spending increases which eventually threaten fiscal sustainability. Ultimately, a trade-off exists between the conflicting wishes for fiscal soundness and true and fair financial reporting in the public sector.

From a research viewpoint, this article develops the political economy of fiscal deficits to take into account the asymmetry of expertise that exists between the various stakeholders in budgeting and accounting. Until now, a lot of attention has been paid to institutional rules and how effective they are in containing fiscal illusions and the resulting spending biases. This paper indicates that it is worthwhile to continue investigating how the technicalities of the budget process, from the planning stage to the reporting stage, make it possible to resolve the uncertainty related to this process and related political conflicts.

## Appendix

**Table 4:** Summary statistics

Variables	Domain	Unit	Mean	Std. dev.	Min	Max
Balance^a^	*R*	CHF/inhabitants	129.914	514.013	− 3151.512	2370.750
Revenues^a^	*R*+	CHF/inhabitants	8435.790	3351.761	4039.267	27755.070
Expenses^a^	*R*+	CHF/inhabitants	8305.876	3333.784	3760.656	26766.070
AD^a^	*R*+	CHF/inhabitants	105.683	250.608	0.000	2819.444
Growth^b^	*R*	Percentage	1.708	2.450	− 7.504	9.635
Unemployment^c^	*R*+	Percentage	2.148	1.723	0.000	7.800
Elderly^d^	*R*+	Percentage	15.020	2.174	10.142	21.756
Leaning^e^	[0–10]	Index	5.550	0.514	4.220	7.057
Fragmentation^e,f^	*R*+	Count	3.437	0.935	1.000	5.000
Concordance^e^	*R*+	Percentage	78.300	22.900	0.000	100.000
Departments^f^	*R*+	Count	7.145	2.174	4.000	13.000
Election^e^	0 or 1	Dummy	0.276	0.447	0.000	1.000
Initiative^d,e,f,g^	[0–6]	Index	4.497	1.138	2.333	6.000
Referendum^d,e,f,g^	[0–6]	Index	3.931	1.181	1.000	6.000
Rules^h^	[0–3]	Index	0.529	0.954	0.000	3.000
Misestimation^a^	*R*	CHF/inhabitants	− 86.072	230.030	− 2145.395	1227.752
*N* = 729						

^a^Annual financial reports of the 26 cantons, compilation by the authors

^b^BAK Basel

^c^State Secretariat for Economic Affairs

^d^Swiss Federal Statistical Office

^e^Année politique suisse

^f^BADAC-Database on Swiss cantons and cities

^g^Finanzdirektorenkonferenz

^h^Luechinger and Schaltegger (2013)

**Table 5:** Covariance between the potentially endogenous covariates and the instruments

Variables	Misestimation	Referendum	Initiative	Rules
Instrument	Misestimation(D1)	Referendum(− 2)	Initiative(− 2)	Rules(− 2)
Covariance	0.526	0.907	0.954	0.872

(− 2) denotes the second lag value of the variable whereas (D1) refers to the first difference of the variable

**Table 6:** Validity of the instruments (2SLS First stage F-stat)

Variables	Misestimation	Referendum	Initiative	Rules
Instrument	Misestimation (D1)	Referendum(− 2)	Initiative(− 2)	Rules(− 2)
*F*-stat	215.41	13.49	22.82	458.47
*p* value	0.000	0.000	0.000	0.000

(− 2) denotes the second lag value of the variable whereas (D1) refers to the first difference of the variable. *F*-stat higher than 16.85 reveals a valid instrument

**Table 7:** Breusch-Pagan/Cook-Weisberg test for heteroskedasticity

	Chi^2^	*p* value
Balance	110.380	0.000
Revenues	375.300	0.000
Expenses	1293.180	0.000

H0: No heteroskedasticity

**Table 8:** Wooldridge test for autocorrelation of order one

	*F*-stat	*p* value
Balance	21.548	0.001
Revenues	36.274	0.000
Expenses	11.735	0.002

H0: No autocorrelation of order one

**Table 9:** Arellano-Bond test for autocorrelation of order one and two

	AR(1)		AR(2)	
	*z*-stat	*p* value	*z*-stat	*p* value
Balance	− 1.730	0.083	0.490	0.626
Revenues	0.530	0.594	1.120	0.263
Expenses	0.140	0.890	0.350	0.728

AR(1) refers to autocorrelation of order one whereas AR(2) denotes autocorrelation of order two. H0: No autocorrelation

**Table 10:** Breusch and Pagan Lagrangian multiplier test for random effects

	Chi^2^	*p* value
Balance	0.390	0.533
Revenues	12.410	0.000
Expenses	4.660	0.031

H0: RE not necessarily appropriate

**Table 11:** Hausman test for random versus fixed effects

	Chi^2^	*p* value
Balance	17.050	0.148
Revenues	28.350	0.005
Expenses	53.780	0.000

H0: Difference in coefficients not systematic

**Table 12:** Variance inflation factor (VIF) for the regressors

Variables	Without FE	Cantonal FE	Cantonal and Time FE
AD(− 1)	1.16	1.39	1.62
Balance(− 1)	1.29	1.42	1.75
Misestimation	1.11	1.26	1.44
Growth	1.07	1.12	2.26
Unemployment	1.62	2.17	9.66
Elderly	1.25	5.37	8.27
Leaning	1.29	2.81	2.99
Fragmentation	1.65	5.85	6.32
Concordance	1.70	7.36	7.53
Departments	1.31	3.11	3.76
Election	1.08	1.14	1.18
Initiative	1.88	35.06	38.01
Referendum	1.85	8.09	8.72
Rules	1.10	3.88	4.82
Mean VIF	1.38	4.93	5.14

Multicollinearity may be an issue when the VIF is equal or higher than 10

## Footnotes

1 The IPSAS apply to every public entity (central government, subnational level of government and municipality). IPSAS terminology is used in the text. When necessary, the French and German equivalent as used in the Swiss cantons are given in footnotes.

2 Finance ministers have personal interests in a sound financial position. They are thus struggling for that. Indeed, their prestige and personal benefits (i.e. re-election perspectives) mainly depend on their ability to achieve that goal (Hallerberg and von Hagen 1999). Dafflon and Rossi (1999) read pointedly that “for a politician seeking re-election, the probability of staying in office may heavily depend on his or her budgetary responsibility”.

7 In addition, a number of Acts of Parliament explicitly require or justify matching revenues (or expenses) for providing specific public services (or collecting specific revenues).

3 See for instance the result of the 2.12.2001 referendum on the implementation of a more stringent budget rule at the central level (debt brake; *Schuldenbremse; Frein aux dépenses*). The proposal was agreed upon in all cantons, with Geneva being the canton where the approval rate was the lowest, but still 74.8% of the voters.

4 *Finanzhaushaltgesetz*, *Loi sur les finances.*

5
*Jahresrechnungsbericht, Rapport sur les comptes annuels.*

6 Quizzically enough, the FMA, and all subsequent amendments to it, is drafted by the cantonal finance minister.

8 Statement of financial performance (also income statement); *Erfolgsrechnung; Compte de résultats*. According to Bergmann (2012), the Neue Zürcher Zeitung (NZZ), a leading Swiss political and business newspaper, almost exclusively mentions information on the statement of financial performance when reporting about the financial situation of local governments.

9 Depreciation charges; *Abschreibungen*; *Amortissements*. To simplify this demonstration, we consider depreciation as the only expense that is purely an accounting charge. Also to simplify, no revenue is purely an accounting revenue. Actually, some other charges do not correspond to disbursement and some revenue do not correspond to receipts, e.g., accrued and deferred liabilities or assets.

10 Additional depreciation charges; *zusätzliche Abschreibungen*; *Amortissements supplémentaires.* AD’s specific budget heading in the detailed general purpose financial report is 332 until year 2008 and 383 since then. Other possibilities for political finessing provided by many FMAs include cookie-jar reserves, reporting capital expenditures as current ones (and vice-versa), false provisions, prefinancing.

11 True depreciation charges (*Abschreibung des Verwaltungsvermögens; Amortissements du patrimoine administratif*) are recognised, according to the chart of accounts, under a specific budget heading (331 until year 2008 and 33 since then).

12 Reported balances have also been cleaned of operations on cookie-jar reserves (*Spezialfinanzierungen; Financements spéciaux*) and truly exceptional operating revenues and operating expenses. The time span of the data set is conditioned by the adoption of the common chart of accounts by the cantons. The chart of account was adopted by some cantons in 1980 already.

13 The implicit Swiss GDP deflator is used as deflator (2012 = 100). Source: Swiss Federal Statistical Office.

14 See Soguel and Ecabert (2015) for more information pertaining additional budget appropriation, spending drift and rebudgeting.

15 The political ideology scale we use to compute leaning was developed by Ladner (2006). The higher the value, the more the government’s political centre of gravity leans to the right.

16 Summary statistics for the controls are presented in Appendix (Table 4). The table also indicates the origin of data used.

17 Results for all the tests are presented in Appendix: Breusch-Pagan/Cook-Weisberg test (Table 7); Wooldridge test for autocorrelation of order one (Table 8); Arellano-Bond test for autocorrelation of order one and two (Table 9); Breusch and Pagan Lagrangian multiplier test for random effects (Table 10); Hausman test for random versus fixed effects (Table 11); and Variance Inflation Factor (VIF) (Table 12).

18 In addition to including two-way fixed effects in the model, operating revenue and operating expenses have been cleaned of exceptional components (e.g. money received extraordinarily from the Swiss National Bank in 2005).

19 See Tables 5 and 6 in Appendix. Instruments proposed in this subsection are used when performing 2SLS and 3SLS (when estimating the simultaneous-equation model).

20 *R* and *E* are also both adjusted for creative accounting operations and exceptional operating revenues and exceptional operating expenses, respectively.

21 We also estimated the model without including time fixed effects. With that specification, in model I, growth becomes positively correlated with the cantonal balance at a 10% level of significance; unemployment correlates significantly (5%) and—as expected—negatively; and elderly turns out to be insignificant.

22 Here again, we estimated the model without including time fixed effects. With that specification, in model I, unemployment, elderly, initiative, and referendum correlate significantly (1 or 5%).

23 According to the mechanics of the cantons’ medium-term financial planning, *t* − 3 is of special interest because that very year is the one that each year newly enters the planning period. Thus, a lag length of 3 years corresponds to reasonable beliefs about the period over which expenses could help predict revenues (and vice-versa).

24 The robustness checks represent a large number of regressions. Due to space constraints, the results are not reported in the article. However, they are available upon request.
